# Tyr1497 in the BRG1 Bromodomain of the SWI/SNF Complex is Critical for the Binding and Function of a Selective BRG1 Inhibitor

**DOI:** 10.1111/jcmm.70518

**Published:** 2025-03-25

**Authors:** Yinan Wang, Chuanhe Yang, Gustavo A. Miranda‐Carboni, Hannah Kelso, Jayaraman Seetharaman, Dong‐Jin Hwang, Duane D. Miller, Lawrence M. Pfeffer

**Affiliations:** ^1^ Department of Pathology and Laboratory Medicine, College of Medicine University of Tennessee Health Science Center Memphis Tennessee USA; ^2^ Department of Medicine, College of Medicine University of Tennessee Health Science Center Memphis Tennessee USA; ^3^ The Center for Cancer Research University of Tennessee Health Science Center Memphis Tennessee USA; ^4^ Department of Pharmacology, Addiction Science, and Toxicology, College of Medicine University of Tennessee Health Science Center Memphis Tennessee USA; ^5^ Department of Pharmaceutical Sciences, College of Pharmacy University of Tennessee Health Science Center Memphis Tennessee USA

**Keywords:** apoptosis, BRG‐1, bromodomain, cell death, DNA damage, glioblastoma, small molecule inhibitor, SWI/SNF

## Abstract

BRG1 and BRM are subunits of the SWI/SNF chromatin remodelling complex, which has DNA‐stimulated ATPase activity and can destabilise histone–DNA interactions. Targeting SWI/SNF is beneficial for treating various tumours, including glioblastoma (GBM). Our research focussed on BRG1 due to its overexpression in GBM. We developed IV‐255, a selective bromodomain (BRD) inhibitor that binds to BRG1 but not BRM. IV‐255 sensitised GBM cells to temozolomide (TMZ), the standard GBM treatment. We identified the binding site of IV‐255 within the BRG1 BRD and found that the Tyr1497 residue is crucial for IV‐255's effect on TMZ‐induced GBM cell death, while Asn1540 is not. Structural analyses confirmed that Tyr1497 is involved in the IV‐255 binding pocket. Mechanistically, IV‐255 increases γH2AX staining in GBM cell nuclei in response to TMZ, indicating an impaired DNA double‐strand break response dependent on Tyr1497. IV‐255 also sensitised GBM cells to TMZ‐induced apoptosis, as shown by PARP and caspase‐3 cleavage, which also requires Tyr1497. In conclusion, Tyr1497 within the BRD of BRG1 is critical for its interaction with IV‐255 and for sensitising GBM cells to TMZ‐induced DNA double‐strand breaks and apoptotic cell death.

**Abbreviations**
BRD: bromodomain
BRG‐1KO: BRG‐1 knockout
CETSA: cellular thermal shift assay
DMSO: dimethyl sulfoxide
GBM: glioblastoma
PARP: Poly (ADP‐ribose) polymerase
TED: therapy enhancing drug
TICD: TMZ‐induced cell death
TMZ: temozolomide

## 1 Introduction

Gliomas are the most common primary brain cancers in adults. While grade IV glioma (GBM, glioblastoma) is the most aggressive and deadliest brain tumour, grade I glioma is the least malignant glioma. The primary treatment modality for GBM is surgical resection combined with adjuvant temozolomide (TMZ) chemotherapy and radiation therapy, which only provides slight improvement in disease course and outcome [1]. The median time for GBM recurrence after surgery is 7 months and the overall prognosis is dismal, with a 5‐year survival of only 5% [2].

The mammalian ATP‐dependent chromatin remodelling SWI/SNF complex is an evolutionarily conserved multi‐subunit complex that regulates gene expression, differentiation, DNA repair and development [3]. The two catalytic subunits, BRM (Brahma) and BRG1 (Brahma‐related gene 1) reposition and/or remodel nucleosomes, which opens or closes chromatin to regulate gene transcription [4]. In adult glioma, BRG1 expression increases with histological tumour grade, with the highest levels found in GBM patients. In contrast, BRM expression is inversely related to tumour grade, with the lowest expression found in GBM patients. BRG1 functions as a tumour suppressor in cancers of the lung, ovaries, skin and blood, with silencing or loss‐of‐function mutations enriched [5, 6, 7, 8, 9]. In contrast, BRG1 has tumour promoting activity in several other cancers, including GBM [7, 10]. Mutations of BRG1 are rarely found in multiple genomic databases of GBM patients [11]. Moreover, we demonstrated that high BRG1 expression selectively localises in GBM patient tumour tissue [12].

The bromodomain (BRD) of BRG1 is an evolutionarily conserved protein–protein interaction module that binds acetyl‐lysine on protein and histone tails [13, 14]. For example, one family member of bromodomain‐containing proteins, BET proteins, regulates the expression of key oncogenes and specific and potent BET inhibitors are now in cancer clinical trials [15, 16, 17]. Thus, BRDs have become attractive targets in cancer. PFI‐3 was originally developed as a highly selective small molecule BRD inhibitor of the BRG1 and BRM subunits of the SWI/SNF complex, which has minimal “off‐target” effects in primary human cells and no evidence of toxicity on the NCI‐60 panel of tumour cell lines [18, 19]. We recently developed analogs of PFI‐3 that we denoted as therapy‐enhancing drugs (TEDs). We found that these TEDs also are not toxic, but they increased the sensitivity of various established GBM cell lines to TMZ and also overcame inherent TMZ resistance in GBM cells [12, 20]. Most interestingly, we identified one such TED IV‐255 whose activity was totally dependent on BRG1 but not BRM expression by a CRISPR/Cas9 gene editing to knockout BRG1 expression in established GBM cell lines.

In this study, we investigated the role of amino acids critical to the inhibitory activity of PFI‐3 [21] to identify the binding site of IV‐255 within the BRG1‐BRD. Through site‐directed mutagenesis of these key residues and restoration of BRG1 expression with these mutant constructs in BRG1 knockout cells, we pinpointed Tyr1497 as essential for the sensitising effect of IV‐255 on TMZ‐induced GBM cell death. However, Asn1540, which was critical to PFI‐3 function, was dispensable to the action of IV‐255. These findings align with *in silico* structural analyses, which revealed that the IV‐255 binding pocket in BRG1 involves Tyr1497 but excludes Asn1540. Mechanistically, IV‐255 enhanced γH2AX staining in GBM cell nuclei in response to TMZ, suggesting that the diminished DNA double‐strand break response was entirely dependent on Tyr1497. Moreover, IV‐255 augmented TMZ‐induced apoptosis in GBM cells, as evidenced by cleavage of Poly (ADP‐ribose) polymerase (PARP) and caspase‐3, both of which also require Tyr1497. In conclusion, we demonstrate that the Tyr1497 residue within the BRD of BRG1 is critical for the interaction with IV‐255 and for its ability to sensitise GBM cells to TMZ‐induced DNA double‐strand breaks and apoptotic cell death.

## 2 Materials and Methods

### 2.1 Biological Reagents and Cell Cultures

LN229 (ATCC, Manassas, VA) and U251 (gift of Dr. James Turkson, City of Hope, CA) GBM cell lines were grown in DMEM containing 10% fetal bovine serum supplemented with penicillin (100 IU/mL) and streptomycin (100 μg/mL) at 37°C with 5% CO_2_. The cells were authenticated by short‐tandem repeat analysis. IV‐255 was designed and synthesised as described in a previous publication [21].

### 2.2 Cell Death and Viability Assays

For death assays, cells were plated into 48 well plates (1 × 10^4^ cells/well), and after three days of drug treatment, the levels of apoptosis in the attached cells were determined according to the instructions using the cell death ELISA^PLUS^ assay (Roche), which measures cytoplasmic histone‐associated DNA fragments. In addition, to determine the effect of various drugs on cell viability and death, the Live/Dead cell viability/cytotoxicity assay (Molecular Probes) was performed according to the manufacturer's protocol. Images were captured on a Zeiss LSM700 laser scanning confocal microscope.

### 2.3 Cellular Thermal Shift Assay (CETSA)

The binding of IV‐255 to the bromodomain of the BRG1 subunit of SWI/SNF was assessed by using a previously described CETSA [21]. BRG1^KO^ GBM cells that express various BRG1 constructs were transduced with a lentivirus encoding an epitope‐tagged BRG1 bromodomain, and BRG1‐tagged expressing cells were treated with IV‐255 (30 μM) or Dimethyl sulfoxide (DMSO) as a vehicle control for 3 h. After heating over a temperature range from 44.5°C to 55.6°C for 5 min, the cells were lysed, placed on ice at 4°C and then immunoblotted for BRG1 or actin.

### 2.4 Restoration of BRG1‐KO Cells

CRISPR/Cas9‐mediated BRG1 knockout of GBM cells was performed as previously described [20]. The two bromodomain (BRD) mutants, Tyr1497Phe (B1) and Asn1540Trp (B2), were made in human SMARCA4 cDNA (GeneCopoeia #GC‐Y3533) using site‐directed mutagenesis (Bio‐red, Q5 Site‐Directed Mutagenesis Kit, NEB #E0554). The wild‐type and BRG1‐BRD mutant (B1, B2 and B3) constructs were cloned to pLenti‐III‐GFP‐N vector (ABM Cat. No. LV031). Lentivirus production and transduction of LN229 and U251 GBM cells were performed as previously described [20].

### 2.5 Immunostaining for γH2AX

GBM cells grown on 8‐well glass chamber slides (Millipore) were treated with IV‐255 (20 μM) with or without TMZ (200 μM) for 48 h and processed for γH2AX immunostaining as previously described [22].

### 2.6 Immunoblotting

Total cell lysates (25 μg) were separated by SDS‐PAGE, immunoblotted with the following antibodies: BRG1 and BRM (Proteintech, Rosemont, IL); PARP, Caspase 3 and cleaved caspase 3 (Cell Signalling Technology, Danvers, MA) and actin (Santa Cruz Biotechnology, Dallas, TX). Following the addition of IRDye800CW goat anti‐mouse IgG or IRDye680 goat anti‐rabbit IgG, blots were visualised on an Odyssey infrared imaging system (LICOR Biosciences, Lincoln, NE) as described previously [23].

### 2.7 Statistical Analyses

At least two independent experiments were performed in duplicate or triplicate, and data are presented as means ± standard deviation (SD). Analysis of variance (ANOVA) and post hoc least significant difference analysis or Student's *t*‐tests were performed.

## 3 Results

### 3.1 Mutations in the BRD Enhance the Sensitivity of GBM Cells to TMZ‐Induced Cell Death

**FIGURE 1:**
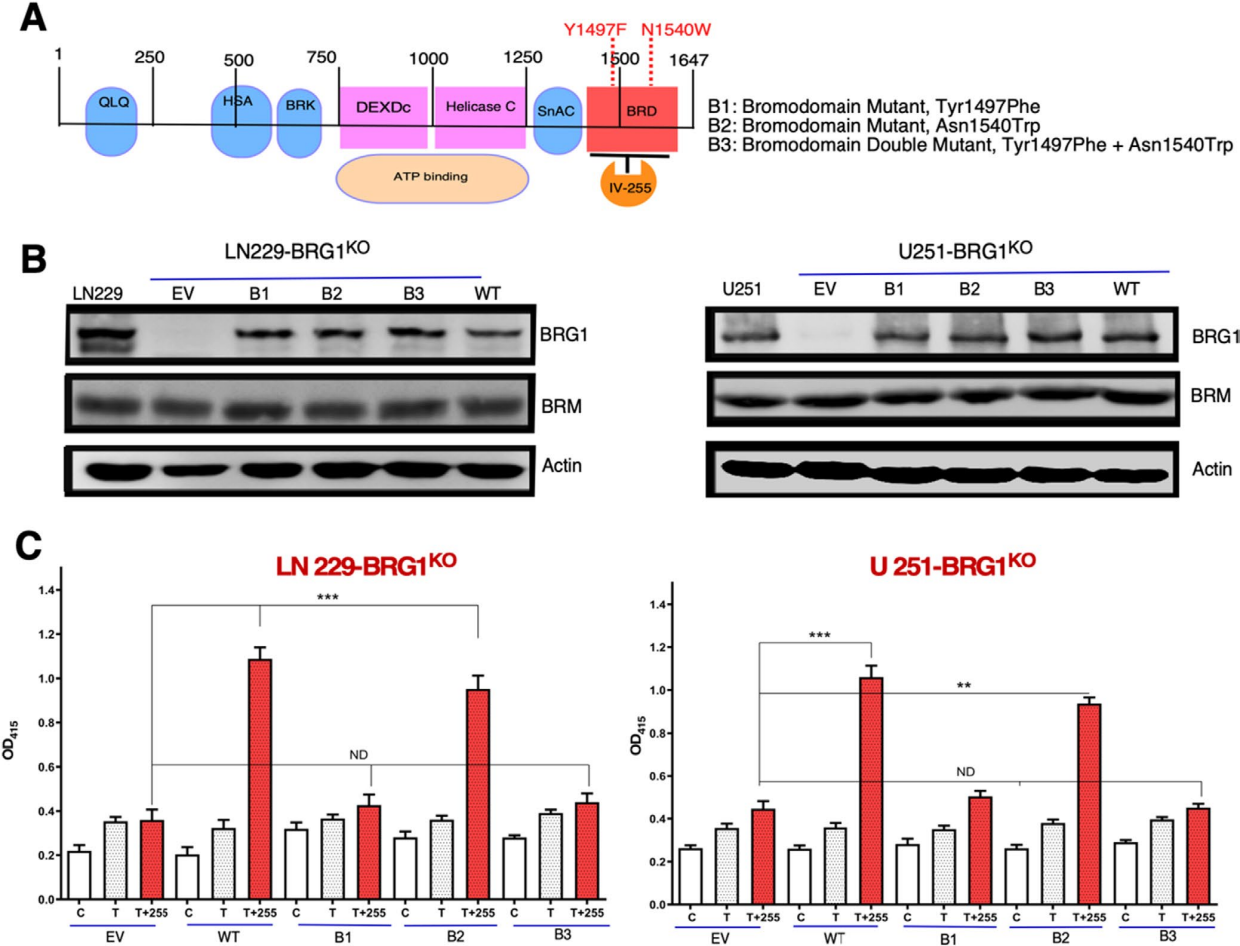
The effects of mutations in the bromodomain of BRG1 on TMZ‐induced cell death. (A) A schematic of the domains in BRG1 showing where the mutations in the BRG1‐BRD were made in Tyr1497 (Y1497F) and Asn1540 (N1540F). (B) Immunoblotting for BRG1 and BRM of lysates of parental (con) and BRG1^KO^ U251 and LN229 GBM cells restored with EV or the BRG1 mutants (B1, B2 and B3). Actin serves as a normalisation control. (C) The various U251 and LN229 BRG1^KO^ cell lines shown in Panel B were treated with IV‐255 (10 μM) alone and with TMZ (200 μM). At this concentration, TMZ alone has only a slight induction of cell death in these two cell lines, as shown previously [22]. Control cells (con) were treated with vehicle (DMSO). A cell death ELISA was performed at 72 h according to the manufacturer's protocol. *** indicates calculated *p* value less than < 0.01.

As shown in the schematic (Figure 1A), we made several mutations in the BRD of BRG1 in amino acids that were previously described [21] to be critical for the epigenetic reader function of the SWI/SNF complex: Tyr1497Phe (B1), Asp1540Trp (B2) and the Tyr1497Phe and Asp1540Trp (B3) double mutant. Both Tyr1497 and Asn1540 read acetylated histone H3K14 [24]. BRG1 knockout LN229 and U251 GBM cells that we previously isolated were transduced with lentiviral vectors encoding these mutations, and cell lysates were immunoblotted. As expected, BRG1^KO^ LN229 and U251 cells did not express BRG1 protein, but transduction with the BRD mutants restored BRG1 expression to parental levels. As evidence of the knockout specificity, BRG1 knockout or restoration with the mutants did not affect the expression of the other catalytic SWI/SNF subunit BRM.

We previously found that IV‐255, a BRG1 selective BRD inhibitor, sensitised GBM cells to TMZ‐induced cell death (TICD) [21]. TMZ is a DNA alkylating agent used to treat GBM patients. Using an ELISA‐based assay, we found that treatment with IV‐255 of BRG1 knockout cells (EV) did not sensitise LN229 or U251 cells to TICD (Figure 1C), demonstrating the requirement of BRG1 for TMZ sensitisation. Restoration of BRG1^KO^ cells with either wild‐type BRG1 (WT) or the B2 mutant (Asp1540Trp mutated) resulted in IV‐255 sensitisation to TICD. In contrast, restoration of BRG1^KO^ cells with either the B1 (Tyr1497Phe) BRD reader site or B3 (mutation of both reader sites) did not show sensitisation to TICD, indicating that the Tyr1497 was critical for IV‐255 TICD sensitisation. Taken together, these results indicate that the Tyr1497 but not Asn1540 in the BRG1 BRD was required for IV‐255 sensitisation of GBM cells to TICD.

### 3.2 The Sensitising Action of IV‐255 on TMZ‐Induced Reduction in GBM Cell Viability Is Dependent on the Tyr1497 Residue in BRG1

**FIGURE 2:**
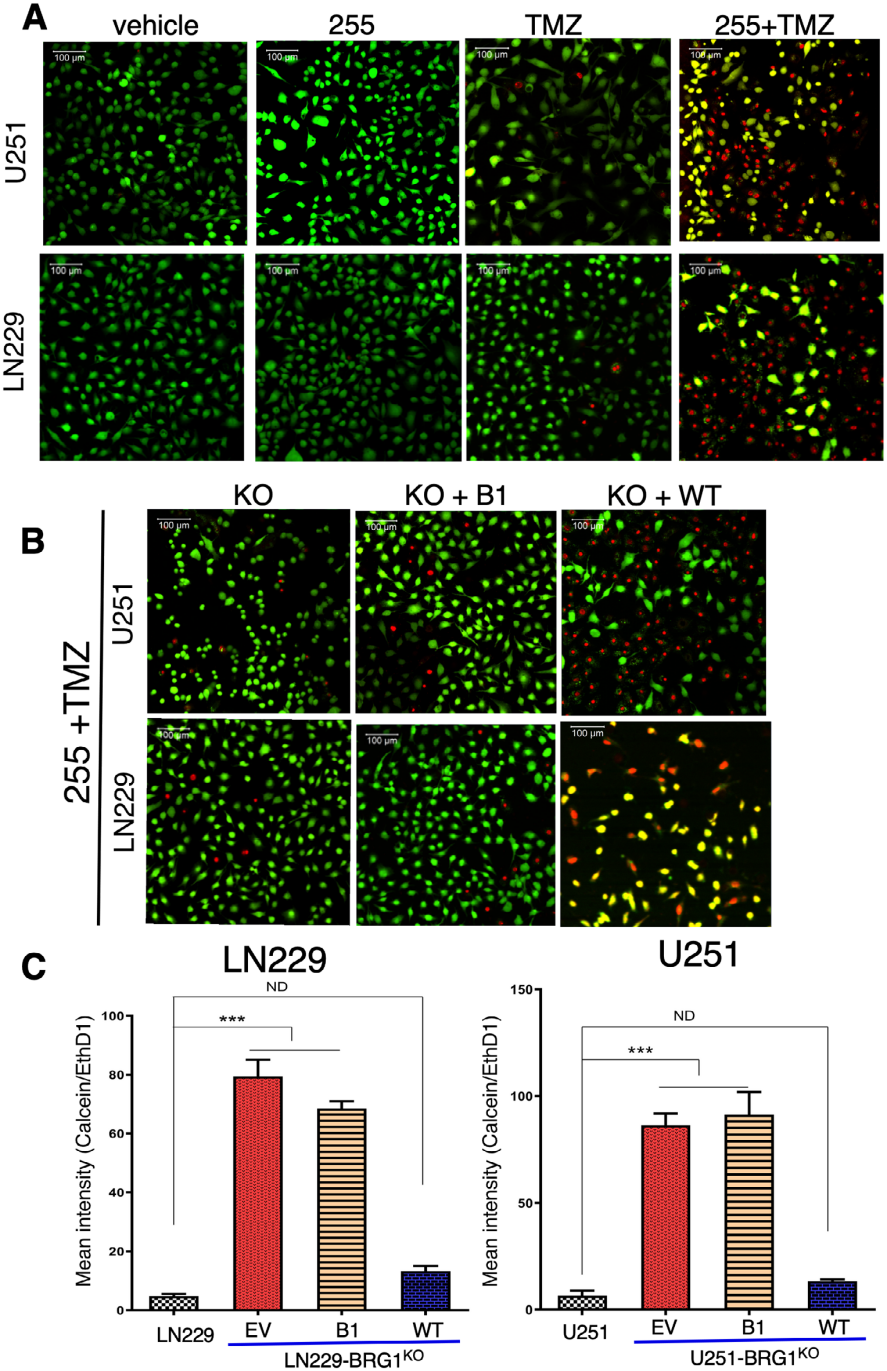
IV‐255 promotes TMZ‐induced reduction of GBM cell viability. (A) parental LN229 and U251 GBM cells were treated with TMZ (200 μM) and IV‐255 (20 μM). After 48 h of treatment, cells were analysed by LIVE/DEAD Viability assay and imaged on a Zeiss LSM 700 confocal microscope. While live cells fluoresce bright green, dead cells fluoresce red‐orange. (B) BRG1^KO^ LN229 and U251 GBM cells restored with either EV, Tyr1497Phe or WT BRG1 were treated with IV‐255 and TMZ and analysed as described in Panel A. (C) The graphs represent the ratio of live (calcein AM) versus dead (ethidium homodimer) staining under the various experimental conditions in panel B. *** indicates calculated *p* value less than < 0.01.

One shortcoming of the ELISA‐based assay is that it does not determine which cells are undergoing death; rather, it identifies the TICD in a cellular population. To address this point, we next employed the live/dead cell fluorescent assay, which distinguishes live cells from dead cells by employing two different fluorescent molecular probes. Exposure of the parental LN229 or U251 GBM cell lines to either IV‐255, a sublethal dose of TMZ (40 μM) or vehicle (DMSO) alone did not induce significant cell death. In contrast, combined treatment of TMZ with IV‐255 in LN229 or U251 cells markedly increased the number of dead (red) cells and reduced the number of live (green) cells in parental cell cultures (Figure 2A). However, treatment with TMZ in combination with IV‐255 of BRG1^KO^ cells restored wild‐type BRG1 markedly induced cell death to a similar extent (Figure 2B). In contrast, treatment of BRG1^KO^ cells restored with the Tyr1497Phe mutation resulted in much fewer dead cells as compared to BRG1^KO^ cells restored with WT‐BRG1. Quantification of these results is shown in Figure 2C. Taken together, these results show that the Tyr1497 reader site plays a critical role in the sensitising action of IV‐255 on TMZ‐induced reduction in GBM cell viability.

### 3.3 Mutations in the BRG1‐BRD Affect Stability of Its Interaction With IV‐255

**FIGURE 3:**
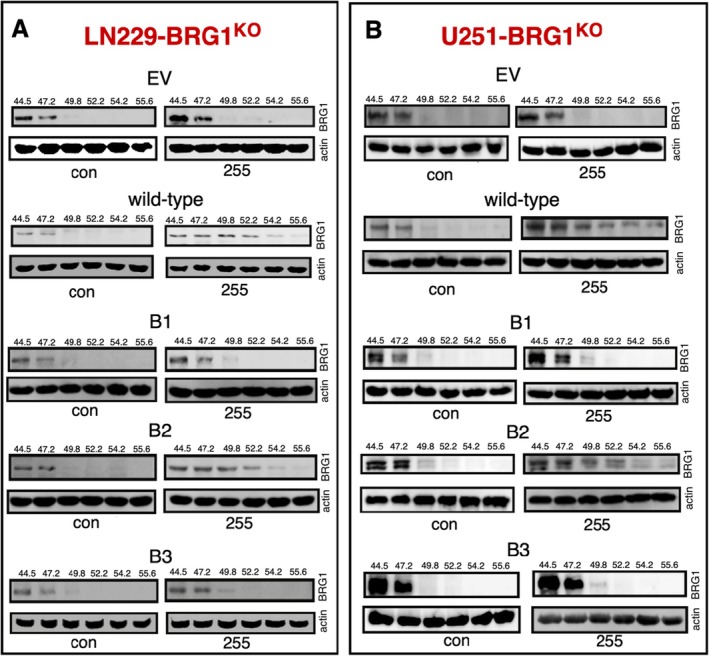
The interaction of IV‐255 with the BRG1 bromodomain in GBM cells expressing BRG1‐BRD mutants. For CETSAs, LN‐229 and U251BRG1^KO^ cells expressing various BRG1 epitope‐tagged BRD mutant constructs were treated with IV‐255 (30 μM) for 3 h. Cells were treated with vehicle (DMSO) as a control. After heating over a temperature range from 44.5°C to 55.6°C for 5 min, the cells were lysed, placed on ice at 4°C and then immunoblotted for BRG1 or Actin.

In previous studies, we employed the cellular thermal shift assay (CETSA) to measure the thermostability of BRD when complexed to IV‐255 and demonstrated that IV‐255 selectively bound to the BRG‐BRD but not to BRM‐BRD [21, 25]. To determine the role of amino acids Tyr1497 and Asn1540 in BRG1‐BRD interaction with IV‐255, BRG1^KO^ LN229 (Figure 3A) and U251 (Figure 3B) cells expressing the various BRG1‐BRD constructs were treated with IV‐255 for 2 h, and then heated over a temperature range of 44.5°C–55.6°C for 5 min. Afterwards, the cells were lysed and immunoblotted for BRG1. IV‐255 significantly increased the thermostability of the BRG1‐BRD in BRG1^KO^ cells expressing wild‐type (WT) BRG1 or the B2 mutant, as evidenced by the band becoming undetectable at 55.6°C (Figure 3A,B). In marked contrast, in BRG1^KO^ cells expressing an empty vector (EV) construct, or the B1 and B3 mutants that have Tyr1497 mutated, the band nearly disappeared at 49.8°C similar to the DMSO control. These results indicate that IV‐255 is selectively bound to the BRG1‐BRD and there is a strict requirement for the Tyr1497 site in the BRG1‐BRD to form a stable complex with IV‐255.

### 3.4 *In Silico* Docking Analysis of the Binding of IV‐255 With the BRG1 Bromodomain

**FIGURE 4:**
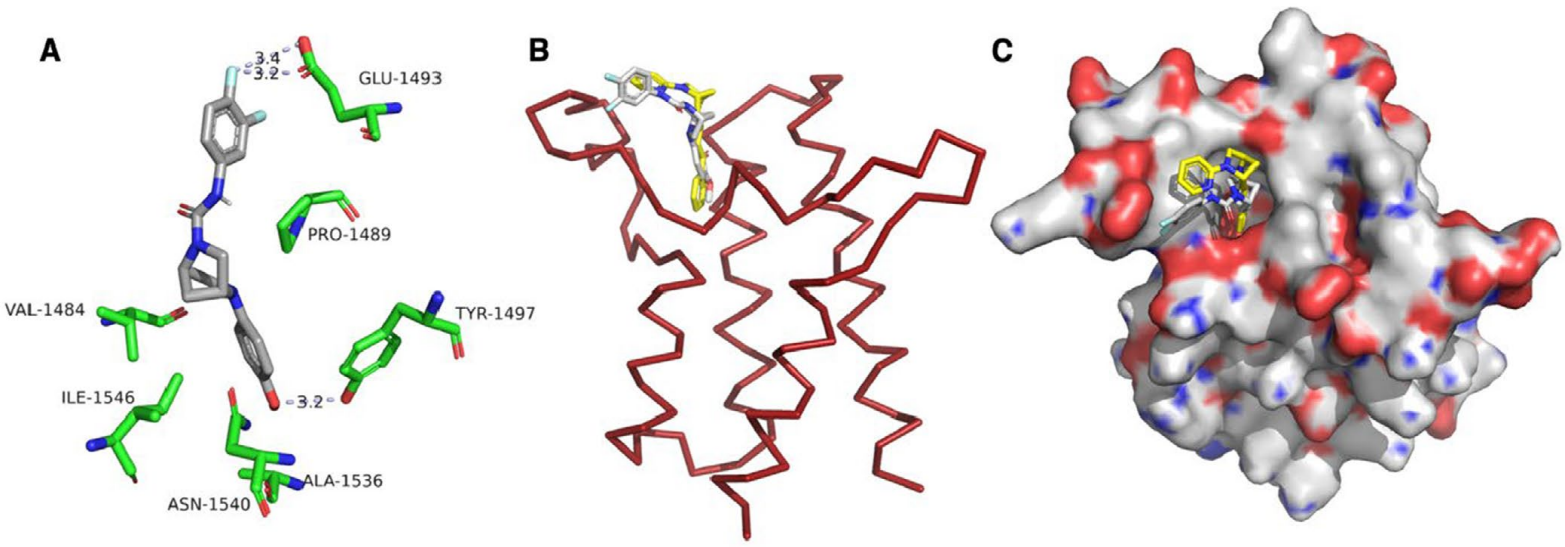
Structural analysis of the docking of IV‐255 to the bromodomain of BRG1. (A) Close‐up view of the binding mode of IV‐255 (grey) with key residues in the BRD of BRG1 (green). (B) Superimposition of IV‐255(grey) and PFI‐3(yellow). BRG1‐BRD is shown in raspberry colour. (C) Surface representation of binding pocket. Blue represents positively charged residues, red represents negatively charged residues and grey represents hydrophobic residues. The compound is bound in the hydrophobic pocket. Panels A–C were prepared by using the Pymol program (http://www.pymol.org/pymol).

Based on the finding that Tyr1497 plays a critical role in the interaction of IV‐255 with the BRG1‐BRD in the sensitisation of GBM cells to TICD, we next performed *in silico* analysis of the potential binding of IV‐255 with the BRG1‐BRD. The cavity analysis reveals four possible binding pockets for the BRD. One of the identified binding pockets matches with an experimentally determined binding pocket of more than 20 crystal structures examined from the Protein Data Bank. This binding pocket is called the Kac pocket, which is a hydrophobic pocket generated by the four helices, forming a deep cavity that is enlarged by two loop regions (ZA and bc loops). IV‐255 was docked at this site within the BRG1‐BRD. The docking was performed with the server CBDock2 [26]. During docking, the water molecules were eliminated because they were displaced by the oxygen atoms in the ligand in experimental crystal structures. The docking results suggest that the hydrogen bond interactions with Tyr1497 and Glu1493, and multiple hydrophobic interactions with Val1484, Ala1536, Phe1485, Leu1488, Phe1489, Glu1493, Tyr1497 and Ile1546 help to stabilise the molecule (Figure 4a). Asn1450 does not make any interactions with compound‐255. The hydrogen bond interaction was lost when Tyr1497 was mutated into Phe1497.

The docking of IV‐255 was then compared with the PFI‐3 structure that was previously experimentally determined (Protein Data Bank 5DKD; crystal structure of the BRD of human BRG1 in complex with the PFI‐3 chemical probe). These two compounds bind in a similar manner and are mainly stabilised by hydrophobic interactions (Figure 4B,C). The ligand PFI‐3 bound with the benzopyrone system is buried deeply in the Kac pocket, forming hydrogen bonds with Tyr1497 and Asn1540. With the exception of these hydrogen bonds, the ligand was stabilised mainly through hydrophobic interactions with Pro1489, Ile1546, Asn1540, Leu1488, Val1484, Phe1485, Val1505 and Ala1536. This shows an important distinction between PFI‐3 which interacts with both Tyr1497 and Asn1540, while IV‐255 interacts with Tyr1497 and Glu1493.

### 3.5 IV‐255 Promotes TMZ‐Induced DNA Damage, Which Is Dependent on the Tyr1497 Site in the BRG1‐BRD

**FIGURE 5:**
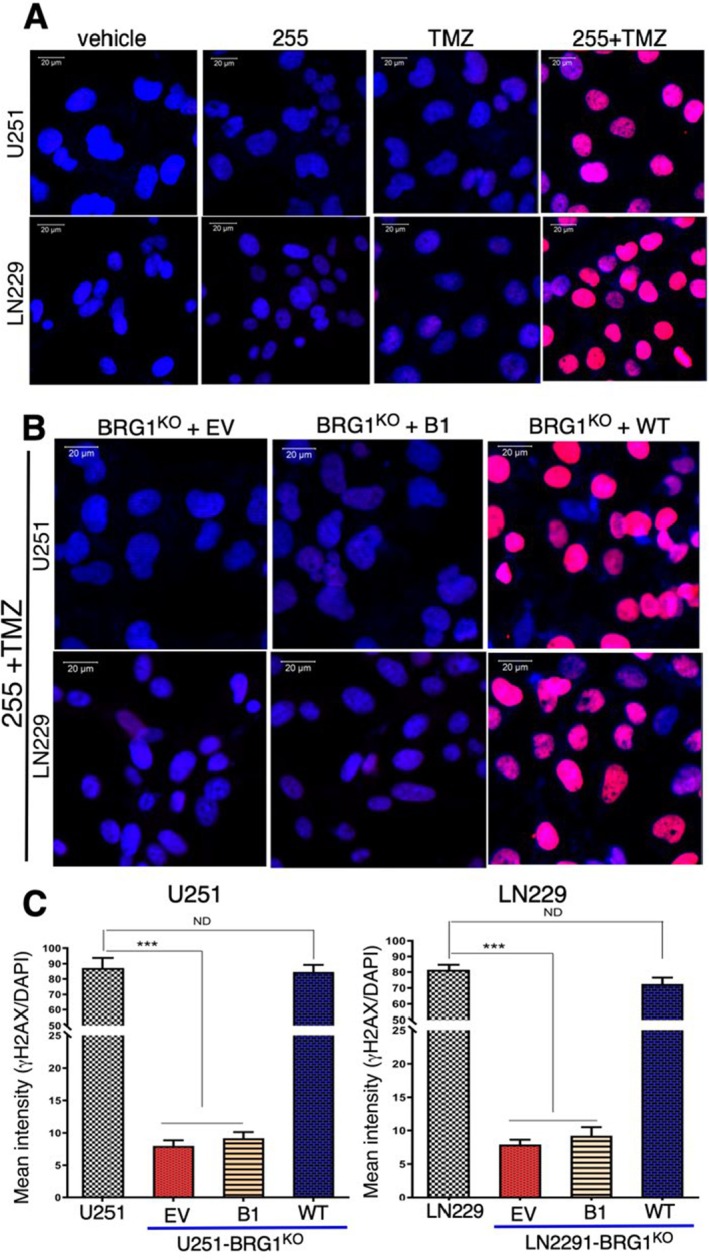
IV‐255 sensitised GBM cells to TMZ‐induced DNA damage. (A) parental LN229 and U251 GBM cells were treated with TMZ (200 μM) and IV‐255 (20 μM). After 48 h of treatment, cells were fixed and immunostained for γH2AX. Immunostaining analysis was performed on a Zeiss LSM 700 confocal microscope and data analysed with a ZEN software focal microscope. (B) BRG1^KO^ LN229 and U251 GBM cells restored with either EV or Tyr1497Phe or WT BRG1 were treated with IV‐255 and TMZ and analysed as described in Panel A. (C) The graphs represent the ratio of γH2AX versus DAPI staining. *** indicates calculated *p* value < 0.01.

TMZ alkylates DNA which leads to DNA damage and subsequent cell death. To investigate the role of IV‐255 in sensitising GBM cells to TMZ‐induced DNA damage, we performed γH2AX immunostaining of LN229 and U251 cell lines. γH2AX represents the phosphorylated form of histone H2AX and functions as a sensitive marker for double‐stranded DNA breaks [27]. A sublethal dose of TMZ induced little γH2AX immunostaining in LN229 and U251 cells. Moreover, IV‐255 also did not induce detectable DNA damage, which is consistent with our finding that IV‐255 is not a cytotoxic drug (Figure 5A). In marked contrast, treatment with IV‐255 in combination with TMZ markedly increased the induction of DNA breaks as evidenced by marked γH2AX staining in both GBM cell lines (Figure 5A).

To examine the functional role of Tyr1497 in the enhancement of TMZ‐induced damage, we also performed γH2AX immunostaining on BRG1^KO^ LN229 and U251 cells expressing wild‐type BRG1 or the Tyr1497Phe mutant construct. As expected, TMZ or IV‐255 alone had no effect on γH2AX staining of GBM cells. In BRG1^KO^ cells restored with wild‐type BRG1, IV‐255 combined with TMZ resulted in intense γH2AX staining (Figure 5B), while γH2AX staining expressing the Tyr1497Phe mutant again demonstrated the requirement for the Tyr1497 residue in the BRD for V‐255 sensitization of GBM cells for TMZ‐induced damage. To quantify the effect on γH2AX staining, we then determined the ratio of γH2AX staining relative to the DAPI nuclear counterstaining. As shown in the graphs in Figure 5C, IV‐255 caused a marked increase in the extent of DNA damage upon TMZ treatment in both TMZ‐sensitive parental U251 and LN229 cells (control) and in BRG1^KO^ expressing wild‐type BRG1. In contrast, there was 50‐ to 100‐fold lower γH2AX staining in BRG1^KO^ cells expressing the Tyr1497Phe mutant or empty vector construct.

### 3.6 IV‐255 Promotes TMZ‐Induced Apoptosis of GBM Cells, Which Is Dependent on Tyr1497 in the BRG1‐BRD

**FIGURE 6:**
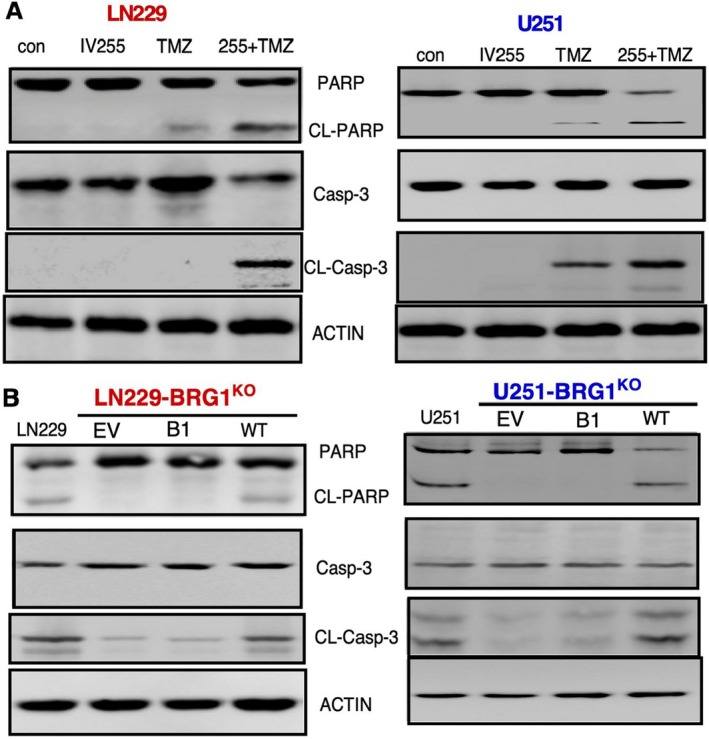
Effect of BRG1‐KD and restoration with WT or the Tyr1497Phe mutant on cellular markers of apoptosis. (A) Parental LN229 and U251 GBM cells were treated with TMZ (200 μM) and IV‐255 (20 μM). After 48 h of treatment, protein lysates were immunoblotted as indicated. (B) BRG1^KO^ LN229 and U251 GBM cells restored with either EV, Tyr1497Phe or WT BRG1 were treated with IV‐255 and TMZ and analysed as described in Panel A.

To examine the underlying mechanism for the sensitisation of GBM cells to TMZ‐induced cell death by IV‐255, parental GBM cells were treated with IV‐255 or TMZ alone, or in combination, and then lysates were prepared and immunoblotted with various markers for apoptosis: PARP, Caspase‐3 and cleaved Caspase‐3. As shown in Figure 6A, while treatment with IV‐255 of either GBM cell alone did not induce PARP or Caspase‐3 cleavage, treatment with TMZ alone only resulted in slight levels of PARP or Caspase‐3 cleavage. Most importantly, treatment of GBM cells with a combination of both IV‐255 and TMZ resulted in a more marked PARP and Caspase‐3 cleavage. Taken together, these findings indicate that IV‐255 enhances the induction of the intrinsic pathway of TMZ‐induced apoptosis in GBM cells. We next examined the role of the Tyr1497 site in this pathway. BRG1^KO^ cells restored with WT or the Tyr1497Phe mutant were treated with the combination of TMZ and IV‐255, and then cells were lysed and immunoblotted as in Figure 6A. As shown in Figure 6B, restoration of BRG1^KO^ cells with WT BRG1 resulted in equivalent levels of PARP and Caspase‐3 cleavage as were observed in parental LN‐229 and U251 cells (Figure 6B). In marked contrast, restoration with the Tyr1497Phe mutant or EV results in little PARP and Caspase‐3 cleavage. The levels of cleaved caspase 3 in control LN229‐BRG1^KO^ and U251‐BRG1^KO^ cells are higher than those in the parental (non‐KO cells), which did not impact our findings. Overall, these results show that the Tyr1497 site in the BRG1‐BRD is required for the sensitization by IV‐255 of TMZ‐induced apoptosis of GBM cells.

## 4 Discussion

The SWI/SNF chromatin remodelling complex is a highly conserved 12‐subunit nucleosome remodelling complex that binds to promoters and enhancers of specific genes to regulate gene expression [28, 29, 30, 31, 32]. Genomic alterations of the BRG1 subunit of SWI/SNF in several human malignancies support its role as a tumour suppressor [3, 11]. In GBM and many other cancers, BRG1 is not mutated but rather overexpressed, suggesting it may also play a pro‐tumorigenic role [12, 20]. Gene deletion and knockdown studies on BRG1 in GBM cells indicate its pro‐tumorigenic role and highlight BRG1 as an attractive therapeutic target in GBM. We demonstrate that, in addition to its canonical role in gene expression, BRG1 is crucial for the DNA damage response, consistent with the finding that BRG1 inactivation leads to reduced phosphorylation of histone H2AX and increased sensitivity to double‐stranded breaks induced by DNA alkylating agents.

Since BRG1 is overexpressed in GBM cells, various approaches have been developed to therapeutically target BRG1 to treat GBM patients [28, 29, 30, 31, 32]. In our studies, we have focused on developing small molecule inhibitors to target the BRG1‐BRD. However, several studies have concentrated on the catalytic activity of BRG1. Small molecule inhibitors of the ATPase activity of BRG1 and BRM have been developed, which repress BRG1/BRM‐dependent gene expression, induce cell differentiation and inhibit the in vitro growth of solid and haematopoietic tumour cells, as well as the in vivo growth of these tumours [28, 29, 30]. For example, FHD‐286, a selective inhibitor of BRG1/BRM, had marked pre‐clinical efficacy in an animal model of acute myeloid leukaemia [31]. In contrast to small molecule inhibitors, PROTAC‐driven degraders hold promise in targeting BRG1 and BRM [32]. Since we found that IV‐255 has high selectivity for the BRG1 bromodomain, it would be of interest to develop a PROTAC based on the IV‐255 structure to target the BRG1 bromodomain as a novel therapeutic strategy for GBM in the future.

PFI‐3 was initially developed as a small‐molecule inhibitor targeting the bromodomains (BRDs) of BRG1 and BRM [19, 36]. Despite initial enthusiasm, its potential as an anticancer drug diminished when it was found to lack cytotoxicity in a broad spectrum of cancer cell lines. However, we discovered that PFI‐3 increases the sensitivity of GBM cells to TMZ‐induced cell death. Building on this finding, we developed structural analogs of PFI‐3, termed TEDs, which further enhance TMZ sensitivity in GBM cells. The two most potent TEDs, IV‐255 and IV‐275, both bind to the BRG1‐BRD. Notably, IV‐255 does not bind to the BRM‐BRD, while IV‐275 does [22]. Furthermore, both IV‐255 and IV‐275 increased TMZ‐induced cell death in BRM knockout (BRM^KO^) GBM cells, yet IV‐255 did not show activity in BRG1 knockout (BRG1^KO^) cells [22]. These findings indicate that IV‐255 is a selective inhibitor of the BRG1‐BRD.

In the present study, we introduced mutations in the BRG1 BRD into BRG1^KO^ GBM cells. Restoration of BRG1 expression with either the wild‐type (WT) or the Asp1540Trp mutant sensitised GBM cells to TMZ in combination with IV‐255. Mutants involving Tyr1497 (B1 and B3) failed to sensitise cells, underscoring the critical role of Tyr1497 in TICD. Live/dead assays confirmed that the combination of IV‐255 and TMZ induced significant cell death in BRG1^KO^ GBM cells restored with WT‐BRG1, but not in cells restored with the Tyr1497Phe mutation in the BRG1‐BRD. This highlights the essential role of Tyr1497 in reducing GBM cell viability in response to IV‐255 and TMZ.

Thermal shift assays showed that IV‐255 stabilises BRG1‐BRD when Tyr1497 is intact, but not in cells with the Tyr1497Phe or double BRG1‐BRD mutant. This demonstrates that Tyr1497 is required for stable IV‐255 binding to BRG1‐BRD. Furthermore, docking analysis revealed that IV‐255 binds the hydrophobic Kac pocket in BRG1‐BRD, forming hydrogen bonds with Tyr1497 and hydrophobic interactions with nearby residues. Unlike PFI‐3, which interacts with both Tyr1497 and Asn1540, the interaction of IV‐255 is exclusive to Tyr1497, explaining its selective sensitivity.

IV‐255 combined with TMZ increased DNA damage in GBM cells, as evidenced by heightened γH2AX staining. This effect depended on Tyr1497 in BRG1‐BRD, as BRG1^KO^ cells with the Tyr1497Phe mutation showed significantly reduced DNA damage. The combination of IV‐255 and TMZ also enhanced markers of apoptosis (cleaved PARP and Caspase‐3) in WT‐BRG1‐expressing cells. Cells expressing the Tyr1497Phe mutant or lacking BRG1 showed minimal apoptosis, confirming the critical role of Tyr1497 in the apoptotic sensitisation by IV‐255.

In the present study, we found that Tyr1497 in BRG1‐BRD is essential for the interaction with IV‐255, enhancing TMZ‐induced DNA damage, cell death and apoptosis in GBM cells. Moreover, we provide evidence through in silico structural evidence that several additional amino acids in the BRD of BRG1 may play critical biological roles in the other functions of this domain, such as its epigenetic reader function. These amino acids that stabilise the interaction of IV‐255 with the BRG1 BRD include Glu1493, Val1484, Ala1536, Phe1485, Leu1488, Phe1489, Glu1493, Tyr1497 and Ile1546. In addition, it will be important in future studies to determine whether Tyr1497 in the BRD undergoes tyrosine phosphorylation, which would enable interactions with transcription factors such as STAT proteins.

## Author Contributions

**Yinan Wang:** data curation (lead), formal analysis (equal), investigation (equal), methodology (equal), validation (equal), visualization (equal), writing – review and editing (equal). **Chuanhe Yang:** conceptualization (equal), data curation (equal), formal analysis (equal), investigation (equal), methodology (equal), supervision (supporting), validation (supporting), writing – review and editing (supporting). **Gustavo A. Miranda‐Carboni:** conceptualization (equal), formal analysis (supporting), supervision (equal), writing – review and editing (equal). **Hannah Kelso:** data curation (equal), formal analysis (equal), investigation (equal), validation (equal), visualization (equal). **Jayaraman Seetharaman:** conceptualization (equal), data curation (equal), formal analysis (equal), investigation (equal), project administration (equal), resources (equal), visualization (equal), writing – original draft (equal), writing – review and editing (equal). **Dong‐Jin Hwang:** investigation (equal), methodology (equal), resources (equal), writing – original draft (equal), writing – review and editing (equal). **Duane D. Miller:** conceptualization (equal), funding acquisition (equal), project administration (equal), resources (equal), supervision (equal), writing – original draft (equal), writing – review and editing (equal). **Lawrence M. Pfeffer:** conceptualization (equal), data curation (equal), formal analysis (equal), funding acquisition (equal), methodology (equal), project administration (equal), resources (equal), supervision (equal), validation (equal), visualization (equal), writing – original draft (equal), writing – review and editing (equal).

## Conflicts of Interest

The authors declare no conflicts of interest.

## Data Availability

The data that support the findings of this study are available from the corresponding author upon reasonable request.

## References

[1] R. Stupp , W. P. Mason , M. J. van den Bent , et al., “Radiotherapy Plus Concomitant and Adjuvant Temozolomide for Glioblastoma,” New England Journal of Medicine 352 (2005): 987–996. 15758009 10.1056/NEJMoa043330

[2] T. S. Surawicz , F. Davis , S. Freels , E. R. Laws, Jr. , and H. R. Menck , “Brain Tumor Survival: Results From the National Cancer Data Base,” Journal of Neuro‐Oncology 40 (1998): 151–160. 9892097 10.1023/a:1006091608586

[3] C. Kadoch and G. R. Crabtree , “Mammalian SWI/SNF Chromatin Remodeling Complexes and Cancer: Mechanistic Insights Gained From Human Genomics,” Science Advances 1 (2015): e1500447. 26601204 10.1126/sciadv.1500447 PMC4640607

[4] K. W. Trotter , H. Y. Fan , M. L. Ivey , R. E. Kingston , and T. K. Archer , “The HSA Domain of BRG1 Mediates Critical Interactions Required for Glucocorticoid Receptor‐Dependent Transcriptional Activation In Vivo,” Molecular and Cellular Biology 28 (2008): 1413–1426. 18086889 10.1128/MCB.01301-07 PMC2258747

[5] M. Imielinski , A. H. Berger , P. S. Hammerman , et al., “Mapping the Hallmarks of Lung Adenocarcinoma With Massively Parallel Sequencing,” Cell 150 (2012): 1107–1120. 22980975 10.1016/j.cell.2012.08.029 PMC3557932

[6] C. Love , Z. Sun , D. Jima , et al., “The Genetic Landscape of Mutations in Burkitt Lymphoma,” Nature Genetics 44, no. 12 (2012): 1321–1325, 10.1038/ng.2468. 23143597 PMC3674561

[7] A. H. Shain and J. R. Pollack , “The Spectrum of SWI/SNF Mutations, Ubiquitous in Human Cancers,” PLoS One 8 (2013): e55119. 23355908 10.1371/journal.pone.0055119 PMC3552954

[8] L. Witkowski , J. Carrot‐Zhang , S. Albrecht , et al., “Germline and Somatic SMARCA4 Mutations Characterize Small Cell Carcinoma of the Ovary, Hypercalcemic Type,” Nature Genetics 46, no. 5 (2014): 438–443, 10.1038/ng.2931. 24658002

[9] F. Le Loarer , S. Watson , G. Pierron , et al., “SMARCA4 Inactivation Defines a Group of Undifferentiated Thoracic Malignancies Transcriptionally Related to BAF‐Deficient Sarcomas,” Nature Genetics 47 (2015): 1200–1205. 26343384 10.1038/ng.3399

[10] C. Kadoch , D. C. Hargreaves , C. Hodges , et al., “Proteomic and Bioinformatic Analysis of Mammalian SWI/SNF Complexes Identifies Extensive Roles in Human Malignancy,” Nature Genetics 45 (2013): 592–601. 23644491 10.1038/ng.2628 PMC3667980

[11] H. C. Hodges , B. Z. Stanton , K. Cermakova , et al., “Dominant‐Negative SMARCA4 Mutants Alter the Accessibility Landscape of Tissue‐Unrestricted Enhancers,” Nature Structural & Molecular Biology 25 (2018): 61–72. 10.1038/s41594-017-0007-3 PMC5909405 29323272

[12] D. Ganguly , M. Sims , C. Cai , M. Fan , and L. M. Pfeffer , “Chromatin Remodeling Factor BRG1 Regulates Stemness and Chemosensitivity of Glioma Initiating Cells,” Stem Cells 36 (2018): 1804–1815. 30171737 10.1002/stem.2909 PMC7427091

[13] P. Filippakopoulos and S. Knapp , “Targeting Bromodomains: Epigenetic Readers of Lysine Acetylation,” Nature Reviews. Drug Discovery 13 (2014): 337–356. 24751816 10.1038/nrd4286

[14] P. Filippakopoulos , S. Picaud , M. Mangos , et al., “Histone Recognition and Large‐Scale Structural Analysis of the Human Bromodomain Family,” Cell 149 (2012): 214–231. 22464331 10.1016/j.cell.2012.02.013 PMC3326523

[15] P. Filippakopoulos , J. Qi , S. Picaud , et al., “Selective Inhibition of BET Bromodomains,” Nature 468, no. 7327 (2010): 1067–1073, 10.1038/nature09504. 20871596 PMC3010259

[16] S. Picaud , D. Da Costa , A. Thanasopoulou , et al., “PFI‐1, a Highly Selective Protein Interaction Inhibitor, Targeting BET Bromodomains,” Cancer Research 73 (2013): 3336–3346. 23576556 10.1158/0008-5472.CAN-12-3292 PMC3673830

[17] E. Nicodeme , K. L. Jeffrey , U. Schaefer , et al., “Suppression of Inflammation by a Synthetic Histone Mimic,” Nature 468 (2010): 1119–1123. 21068722 10.1038/nature09589 PMC5415086

[18] B. S. Gerstenberger , J. D. Trzupek , C. Tallant , et al., “Identification of a Chemical Probe for Family VIII Bromodomains Through Optimization of a Fragment Hit,” Journal of Medicinal Chemistry 59, no. 10 (2016): 4800–4811, 10.1021/acs.jmedchem.6b00012. 27115555 PMC5034155

[19] O. Fedorov , J. Castex , C. Tallant , et al., “Selective Targeting of the BRG/PB1 Bromodomains Impairs Embryonic and Trophoblast Stem Cell Maintenance,” Science Advances 1 (2015): e1500723. 26702435 10.1126/sciadv.1500723 PMC4681344

[20] Y. Wang , C. H. Yang , A. P. Schultz , M. M. Sims , D. D. Miller , and L. M. Pfeffer , “Brahma‐Related Gene‐1 (BRG1) Promotes the Malignant Phenotype of Glioblastoma Cells,” Journal of Cellular and Molecular Medicine 25 (2021): 2956–2966. 33528916 10.1111/jcmm.16330 PMC7957270

[21] C. Yang , Y. Wang , M. M. Sims , Y. He , D. D. Miller , and L. M. Pfeffer , “Targeting the Bromodomain of BRG‐1/BRM Subunit of the SWI/SNF Complex Increases the Anticancer Activity of Temozolomide in Glioblastoma,” Pharmaceuticals (Basel) 14, no. 9 (2021): 904, 10.3390/ph14090904. 34577604 PMC8467157

[22] C. Yang , Y. He , Y. Wang , et al., “Next‐Generation Bromodomain Inhibitors of the SWI/SNF Complex Enhance DNA Damage and Cell Death in Glioblastoma,” Journal of Cellular and Molecular Medicine 27, no. 18 (2023): 2770–2781, 10.1111/jcmm.17907. 37593885 PMC10494295

[23] C. H. Yang , J. Yue , S. R. Pfeffer , et al., “MicroRNA‐21 Promotes Glioblastoma Tumorigenesis by Down‐Regulating Insulin‐Like Growth Factor‐Binding Protein‐3 (IGFBP3),” Journal of Biological Chemistry 289 (2014): 25079–25087. 25059666 10.1074/jbc.M114.593863 PMC4155674

[24] B. Vangamudi , T. A. Paul , P. K. Shah , et al., “The SMARCA2/4 ATPase Domain Surpasses the Bromodomain as a Drug Target in SWI/SNF‐Mutant Cancers: Insights From cDNA Rescue and PFI‐3 Inhibitor Studies,” Cancer Research 75 (2015): 3865–3878. 26139243 10.1158/0008-5472.CAN-14-3798 PMC4755107

[25] Y. He , C. Yang , Y. Wang , et al., “Novel Structural‐Related Analogs of PFI‐3 (SRAPs) That Target the BRG1 Catalytic Subunit of the SWI/SNF Complex Increase the Activity of Temozolomide in Glioblastoma Cells,” Bioorganic & Medicinal Chemistry 53 (2022): 116533. 34863065 10.1016/j.bmc.2021.116533

[26] Y. Liu , X. Yang , J. Gan , S. Chen , Z. X. Xiao , and Y. Cao , “CB‐Dock2: Improved Protein‐Ligand Blind Docking by Integrating Cavity Detection, Docking and Homologous Template Fitting,” Nucleic Acids Research 50, no. W1 (2022): W159–W164, 10.1093/nar/gkac394. 35609983 PMC9252749

[27] H. Yang , N. Asaad , and K. D. Held , “Medium‐Mediated Intercellular Communication Is Involved in Bystander Responses of X‐Ray‐Irradiated Normal Human Fibroblasts,” Oncogene 24 (2005): 2096–2103. 15688009 10.1038/sj.onc.1208439

[28] G. R. Hoffman , R. Rahal , F. Buxton , et al., “Functional Epigenetics Approach Identifies BRM/SMARCA2 as a Critical Synthetic Lethal Target in BRG1‐Deficient Cancers,” Proceedings of the National Academy of Sciences of the United States of America 111, no. 8 (2014): 3128–3133, 10.1073/pnas.1316793111. 24520176 PMC3939885

[29] J. P. N. Papillon , K. Nakajima , C. D. Adair , et al., “Discovery of Orally Active Inhibitors of Brahma Homolog (BRM)/SMARCA2 ATPase Activity for the Treatment of Brahma Related Gene 1 (BRG1)/SMARCA4‐Mutant Cancers,” Journal of Medicinal Chemistry 61, no. 22 (2018): 10155–10172, 10.1021/acs.jmedchem.8b01318. 30339381

[30] F. J. de Miguel , C. Gentile , W. W. Feng , et al., “Mammalian SWI/SNF Chromatin Remodeling Complexes Promote Tyrosine Kinase Inhibitor Resistance in EGFR‐Mutant Lung Cancer,” Cancer Cell 41 (2023): 1516–1534.e9. 37541244 10.1016/j.ccell.2023.07.005 PMC10957226

[31] W. Fiskus , J. Piel , M. Collins , et al., “BRG1/BRM Inhibitor Targets AML Stem Cells and Exerts Superior Preclinical Efficacy Combined With BET or Menin Inhibitor,” Blood 143, no. 20 (2024): 2059–2072, 10.1182/blood.2023022832. 38437498 PMC11830987

[32] R. B. Kargbo , “SMARCA2/4 PROTAC for Targeted Protein Degradation and Cancer Therapy,” ACS Medicinal Chemistry Letters 11 (2020): 1797–1798. 33062156 10.1021/acsmedchemlett.0c00347 PMC7549100

